# Infestation by Phorid Flies Disrupts Behavior and Immune Function in Honey Bees Monitored by Radio-frequency Identification

**DOI:** 10.1007/s13744-025-01352-9

**Published:** 2026-02-19

**Authors:** Gloria Ruiz-Guzmán, Oliverio Delgado-Carrillo, Francisco J. Balvino-Olvera, María de Jesús Aguilar-Aguilar, Violeta Patiño-Conde, Paulo de Souza, Ulises Olivares-Pinto, Mauricio Quesada

**Affiliations:** 1 Laboratorio Nacional de Análisis y Síntesis Ecológica (LANASE), Escuela Nacional de Estudios Superiores (ENES) Unidad Morelia, Universidad Nacional Autónoma de México (UNAM), Morelia, Michoacán México; 2 Posgrado en Ciencias Biológicas, Unidad de Posgrado, Ciudad Universitaria, Coyoacán, México; 3 Unidad Académica de Ecología y Biodiversidad Acuática, Instituto de Ciencias de Mar y Limnología, Universidad Nacional Autónoma de México (UNAM), Ciudad de Mexico, México; 4 School of Engineering, Edith Cowan University, Perth, WA 6027 Australia; 5 Escuela Nacional de Estudios Superiores (ENES) Unidad Juriquilla, Universidad Nacional Autónoma de México (UNAM), Campus Juriquilla, Querétaro, México; 6 Instituto de Investigaciones en Ecosistemas y Sustentabilidad (IIES), Universidad Nacional Autónoma de México (UNAM), Campus Morelia, Morelia, Michoacán México

**Keywords:** Colony losses, RFID monitoring, *Apis mellifera*, *Megaselia*, Behavior, Immune system

## Abstract

Honey bees (*Apis mellifera* Linnaeus) are important pollinators in natural and agricultural ecosystems and play a key role in global food production. However, colonies are increasingly affected by multiple stressors, including emerging parasites, which can compromise their health and performance. Among these, phorid flies (Diptera: Phoridae) have been observed in association with weak or collapsing honey bee colonies in various regions in Mexico, yet their effects on honey bee behavior and immune function under field conditions remain poorly understood. In this study, we investigated the impact of a phorid fly infestation on the behavior, survival, and immune defense of *A. mellifera* under natural conditions. Using radio-frequency identification (RFID) technology, we monitored the daily activity of individually tagged bees from a healthy hive and a naturally infested hive. Additionally, we assessed the prevalence of coinfections and measured immune responses including prophenoloxidase (proPO), phenoloxidase (PO), lytic activity, and hemocyte counts. Our results showed that phorid fly infestation was associated with a reduction in the frequency and duration of foraging trips, nocturnal activity, and significant changes in immune function, particularly elevated proPO/PO activity and reduced total hemocyte counts. No significant differences were observed between adult and pupal stages in immune parameters, suggesting a consistent physiological response across developmental stages. While only one hive was infested and ultimately collapsed, these findings provide evidence that phorid flies can disrupt key behavioral and immune processes in honey bees. Further studies are needed to determine the broader implications of phorid infestations for colony health and resilience.

**Supplementary Information:**

The online version contains supplementary material available at 10.1007/s13744-025-01352-9.

## Introduction

Over recent decades, bees and other pollinators have faced significant global declines, threatening pollinator-dependent ecosystems and food crops (Ashworth et al. 2009; Novais et al. 2016; Simmons et al. 2019; Hristov et al. 2020; Balvino-Olvera et al. 2024). Currently, managed bees continue to experience colony losses, which remain poorly understood in various parts of the world, including the tropics (vanEngelsdorp et al. 2009; Currie et al. 2010; Brodschneider et al. 2018; Gray et al. 2023; Medina-Flores et al. 2023; Lamas et al. 2024; Requier et al. 2024). These losses have been attributed to multiple factors including habitat degradation, land use change, pesticide exposure, extreme weather events, and parasitism, which are believed to synergistically alter bee performance (vanEngelsdorp et al. 2009; Perry et al. 2015; Potts et al. 2016; Roy et al. 2018).

Honey bees exhibit behaviors that significantly increase their vulnerability to parasites. Specifically, worker bees, the most numerous and active members of a colony, perform a wide range of age-related tasks essential for colony survival, including brood care, nest maintenance, defense, and foraging. Foraging bees, in particular, frequently interact with other colonies (Oliveira et al. 2021) and drone congregation areas (Ayup et al. 2021), thus increasing their exposure to parasites. This not only makes them susceptible to infection but also the likelihood that foragers act as vectors within the colony (Graystock et al. 2015; Nunes-Silva et al. 2019). Consequently, parasitic pressure can alter essential worker functions such as foraging activity, nest-related tasks, and defense-related behavior, and may compromise overall colony performance, particularly under conditions of limited floral resources (Polatto et al. 2014; Koch et al. 2017).

To counter parasites and pathogens, honey bees rely on a suite of behavioral and immune defenses. Behavioral defenses operate at the colony level, including social immunity mechanisms that mitigate pathogen spread and damage (Cremer 2019). In parallel, the immune response involves both cellular (e.g., hemocytes mediating phagocytosis and encapsulation) and humoral components (e.g., antimicrobial peptides, lysozymes, and the prophenoloxidase system) that target a wide range of invaders including viruses, bacteria, fungi, and parasitic arthropods (Gillespie et al. 1997; Kanost et al. 2004; Tang et al. 2006). Among these parasites, phorid flies have been recognized as a significant mortality factor in stingless bee colonies in Central America (Robroek et al. 2003) and are increasingly reported as emerging threats to honey bee health across several regions, including the USA, Italy, Saudi Arabia, Egypt, Algeria, and Central America (Core et al. 2012; Dutto and Ferrazzi 2014; Khattab and El-Hosseny 2014; Menail et al. 2016; Cham et al. 2018; Mohammed 2018; Dias de Freitas et al. 2023). Although most studies focus on the presence of these flies within honey bee colonies (see Table 1), little is known about their actual impact on colony health and defense mechanisms.

**Table 1:** Reviewed studies (from 2010 to 2024) about the parasitic behavior of phorid flies observed in honey bees

Region	Phorid fly species	Bee species	Parasitic behavior	Effect of infection	Fly origin	Detection method	Parasitism percentage	Coinfection	Bee defense trait	Reference
USA (California, South Dakota)	*Apocephalus borealis*	*Apis mellifera*	Parasitoid	Behavioral manipulation, disorientation, nocturnal abandonment, death	Field/lab	Morphological, DNA barcode (COI), rRNA	Up to 77% of sites	*Nosema ceranae*, DWV, BQCV	/	Core et al. 2012
Italy (Piedmont)	*Megaselia rufipes*	*Apis mellifera*	Parasitoid	Limited movement	Field	Morphological	23% of adults	DWV, *Varroa destructor*	/	Dutto and Ferrazzi 2014
Egypt	*Apocephalus borealis*	*Apis mellifera*	Parasitoid	Abnormal behavior, abandonment	Field	Morphological	Up to 37% of colonies	/	/	Khattab and El-Hosseny 2014
Algeria (Annaba)	*Megaselia scalaris*	*Apis mellifera intermissa*	Parasitoid	/	Field	RT-qPCR	/	DWV in bees and flies	/	Menail et al. 2016
Italy (central–south)	*Megaselia scalaris*	*Apis mellifera*	Parasitoid	/	Field	Morphological	Up to 80% of apiaries	/	/	Ricchiuti et al. 2016
Cameroon	*Megaselia scalaris*	*Apis mellifera*	Parasitoid	/	Field	Morphological, DNA barcode, qPCR	9.2% of colonies	/	/	Cham et al. 2018
Slovakia	*Megaselia* spp.	*Apis mellifera*	Parasitoid, beehive parasite (on eggs and larvae in capped cells)	/	Field	Morphological	/	Fungi	/	Sabo et al. 2020
South Korea	*Apocephalus borealis*	*Apis mellifera*	/	/	Field	RT-qPCR	1.71% of adults	*Aspergillus flavus*, ABPV, KBV	/	Truong et al. 2023

The slash indicates missing information in each case

In Mexico, beekeepers have observed phorid flies during episodes of high colony mortality. In 2016, for example, the Comarca Lagunera region in northern Mexico reported the loss of approximately 3066 of 6256 hives. Similar events occurred in central (San Luis Potosí, Querétaro), southern (Oaxaca, Chiapas), and southeastern (Campeche) regions, based on personal reports from beekeepers. While the parasitic behavior of *Apocephalus borealis* is well documented (Core et al. 2012), other phorid species have also been observed infesting weak or decaying *A. mellifera* colonies (Disney 2008; Dutto and Ferrazzi 2014), and occasionally even in apparently healthy hives (Ricchiuti et al. 2016). However, no field studies have evaluated how these infestations affect bee behavior and immune function under natural conditions.

In this study, we evaluated the effect of phorid fly infestation on honey bee behavior and immune responses under field conditions. While conducting routine monitoring in ten apiaries in Mexico as part of the Global Initiative for Honey Bee Health (GIHH), led by the Australian Commonwealth Scientific and Industrial Research Organisation, we identified a case of phorid infestation in one hive (H2-infested) adjacent to a healthy reference hive (H1-healthy). We intensified monitoring of these two colonies by equipping individual worker bees with radio-frequency identification (RFID) tags to record their activity patterns (Susanto et al. 2018), capturing detailed records of incoming and outgoing movements. We evaluated the impact of phorid infestation by comparing survival, behavior, and immune response between hives. Immune parameters included humoral (phenoloxidase, prophenoloxidase, and lytic activity) and cellular (hemocyte counts) components, which reflect physiological condition of bees and their investment in defense (Hill 2011). We predicted that the H2-infested hive would show elevated immune activity and reduced survival, despite being managed under the same conditions as the H1-healthy hive. To control for confounding factors, we also screened for prevalent pathogens such as *Ascosphaera apis*, *Melissococcus plutonius*, and *Vairimorpha* (*Nosema*) *ceranae* in both colonies. To our knowledge, this is the first field-based study to explore the phorid fly infestation on behavior and defense in *A. mellifera* under a natural coinfection scenario.

## Material and Methods

### Study Site, Observations, and Sampling

This study was conducted in July and August 2016 at an apiary located in Los Tigrillos, Michoacán (19°41′58.68″ N, 101°0′2.46″ W) within a pine-oak forest at an elevation of 2023 m.a.s.l. Two hives were intensively monitored: H1 represented the healthy hive not infested by flies (and no other parasites); and H2, the hive colonized by phorid flies. Both hives were located nearby in the same apiary. The H1-healthy hive was monitored for eight consecutive weeks, while the H2-infested hive was monitored for five consecutive weeks, until it collapsed. In the H2-infested hive, phorid eggs, larvae, and pupae were directly collected from naturally parasitized bees at different developmental stages. Adult worker bees and pupae (aged 12–14 days) were sampled from both hives. The brood frames and the collected samples were transported to the laboratory and stored at − 70 °C until use.

### Taxonomic and Molecular Classification of Phorid Flies

Phorid flies were classified based on morphological traits described by Disney (1994, 2008) and by molecular barcoding using the cytochrome oxidase I (COI) DNA sequence that distinguishes between Phoridae species (Boehme et al. 2010). The genomic DNA of 21 individual adult flies was extracted using Doyle’s (1991) protocol, modified by adding 1% PVP in the extraction buffer. Samples were incubated with 2.5 μL Proteinase K (20 mg/mL per 500 μL extraction buffer), for 3 h at 65 °C with constant agitation. The COI gene was amplified in a 12.5-μL reaction using the QIAGEN Multiplex PCR Kit with primers LCO1490 and HCO2198 (Folmer et al. 1994), and the PCR conditions adapted from Boehme et al. (2010), including an initial activation step of 15 min at 95 °C for the HotStarTaq DNA polymerase. All PCR reactions were performed on a Mastercycler® nexus gradient (Eppendorf). PCR products were analyzed using the QIAxcel Advanced system-(QIAGEN), where a ~ 740-bp fragment was determined using the Screening Kit (QIAxcel-QIAGEN) and method OM400 described in the *QIAxcel DNA Handbook* (sample uptake 10 s at 5 kV, separation 400 s at 6 kV). Amplicons were sequenced in both directions by Macrogen (Seoul, Korea), and assembled using Sequencher.

### DNA Extraction and Detection of Parasites

The thorax of adult bees and entire pupae were homogenized in liquid nitrogen in pools of five individuals with adults and pupae processed separately. In total, we obtained six pools of adults and seven pools of pupae from the H1-healthy hive, and 13 pools of adults and 12 pools of pupae from the H2-infested hive. The DNA was extracted from each pool using the cetyltrimethylammonium bromide (CTAB) method with 1% PVP and 0.01% β-mercaptoethanol, followed by incubation with 2.5 μL Proteinase K for 1 h at 65 °C (600 rpm). The extracted DNA was quantified using an Eppendorf Biospectrometer® basic. The prevalence of parasites was assessed as the proportion of positive pools, including *Vairimorpha* *ceranae* in adults, *Melissococcus plutonius*, and *Ascosphaera apis* in pupae. Each pathogen was targeted with a single diagnostic primer (see Table 2 for references and PCR conditions).

**Table 2:** Prevalence of the parasites *Ascosphaera apis*, *Melissococcus plutonius*, and *Vairimorpha* *ceranae* expressed as the percentage of positive pools in adult worker bees and pupae of the H1-healthy hive and the H2-infested hive

Parasite prevalence (% of positive pools)					
Hive	Age	No. of pools (No. of bees)	*Ascosphaera apis*^a^	*Melissococcus plutonius*^a^	*Vairimorpha* *ceranae*^b^
H1-healthy	Pupae	7 (35)	0	0	-
	Adults	6 (30)	-	-	100
H2-infested	Pupae	12 (60)	0	0	-
	Adults	13 (65)	-	-	0

The dashes mean that those parasites were not tested at that developmental stage. Primer references: ^a^Garrido-Bailón et al. (2013); ^b^Martín-Hernández et al. (2007).

### Bee Activity

**Fig. 1:**
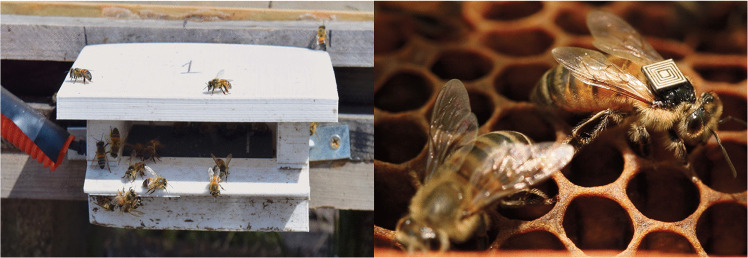
Left: RFID reader installed at the entrance of each hive for data collection. Right: A worker honey bee with an RFID tag attached on the top of the thorax. As the bee passes through the only hive entrance, the tag is detected and the bee’s ID, date, and time are recorded in daily CSV files

As part of the Global Initiative for Bee Health (GIHH) implemented by the Australian Commonwealth Scientific and Industrial Research Organisation (CSIRO; https://www.csiro.au/en/news/All/News/2015/August/Honey-Bee-Health), the activity of the bees (incoming and outgoing records) was monitored using radio-frequency identification (RFID) tags that were glued to the thorax of adult bees with superglue (see: shellac has produced better results, Toppa et al. 2020). The tag’s size was 2.5 × 2.5 × 0.4 mm; each tag weighed 2.4 mg (Hitachi Chemical, Japan) and allowed the recording of a unique bee identification number in hexadecimal format (de Souza et al. 2018). One hundred tags per hive (H1-healthy and H2-infested) were tagged weekly over eight consecutive weeks. An RFID reader (USB Desktop RU-824) installed at each hive’s entrance recorded the daily tags’ signals, capturing data on each individual bee’s movements (Fig. 1).

The RFID system was autonomously controlled by an Edison Intel Atom Processor microcomputer (1 GB memory, 5 GB storage, Wi-Fi, and Bluetooth enabled), powered by solar cells. Data were wireless downloaded weekly. The system recorded each tagged bee’s unique ID upon passing through the reader. Activity was analyzed according to four behavior categories suggested by Susanto et al. (2018): (1) By the entry (BTE) or successive readings of the same bee within less than 3 min indicates that this particular bee is near the RFID reader; (2) short mission (SM) or the individual bee engaged in an activity of short duration (between 3 and 6 min), for example, making orientation or defecation flights and inspecting the hive surroundings; (3) foraging (FG) or the individual bee engaged in activities to search for or exploit food/water sources (6 min to more than 6 h), example bee roles undertaking such activities are scout, exploiter, recruit, and water carrier; and (4) departed bee (DB), the last detection of a bee during its lifetime. Daily activity was categorized according to the day and nighttime it was recorded: (1) sunrise, from 7 a.m. to 12 a.m.; (2) sunset, from 12 a.m. to 7 p.m. (Susanto et al. 2018); (3) night, from 7 p.m. to 12 p.m.; and (4) midnight, from 12 p.m. to 7 a.m. The activity duration was considered as the difference in time between one reading and the subsequent reading.

### Immune Response and Health Parameters

A sample of the homogenized tissue of adults and pupae was used to assess immune system parameters including prophenoloxidase activity (proPO), phenoloxidase activity (PO), and lytic activity (LA). Each sample (30 mg) was homogenized in 400 μL of phosphate buffered saline (PBS, pH 7.4, Sigma), vortexed, and centrifuged (17,000 × *g* for 15 min). The supernatant was used for subsequent protein and immune analyses (Nicoletti et al. 2020). Total protein content was also measured as a proxy of nutritional condition (Contreras-Garduño et al. 2007; Lee et al. 2008; Nicoletti et al. 2020; Burciaga et al. 2023).

#### Prophenoloxidase (proPO) and Phenoloxidase (PO) Activities

The PO activity in adults and pupae from each hive was quantified by the oxidation of *L*-dihydroxyphenylalanine (L-DOPA; Sigma-Aldrich) to dopachrome (Contreras-Garduño et al. 2007). The proPO is the inactive zymogen precursor to PO, an important enzyme to innate immune function that results in the formation of melanin (Wilson-Rich et al. 2009), which leads to cuticle sclerotization, wound healing, and cellular defense responses against infectious agents (Ratcliffe et al. 1984; Söderhäll and Cerenius 1998; Chan et al. 2009; Laughton and Siva-Jothy 2011). For each reaction, 50 μL of sample, 40 μL PBS, and 10 μL of L-DOPA solution (4.0 mg/mL) were added to a 96-well plate. Absorbance was read every 3 min for 60 min at 490 nm in a Multiskan GO Microplate reader (Thermo Scientific). In each well of a 96-well microplate, 50 μL of the sample, 40 μL of PBS, and 10 μL of L-DOPA solution were added. In total, 50 μL of PBS was added to the control wells instead of the sample. Each plate was read at 490 nm every 3 min for 60 min in a Multiskan GO Microplate Spectrophotometer (Thermo Scientific) after 15 min of incubation at room temperature. Due to the cytotoxic nature of the products in the melanization cascade, PO is commonly stored in the inactive precursor proPO and activated naturally following the recognition of foreign compounds or artificially by using chymotrypsin that activates PO production (Ratcliffe et al. 1984; Söderhäll and Cerenius 1998; Laughton and Siva-Jothy 2011). Thus, proPO activity was activated using 2 μL of ɑ-chymotrypsin (0.25 mg/mL) as an artificial activator (Ratcliffe et al. 1984; Söderhäll and Cerenius 1998; Eleftherianos et al. 2008; Laughton and Siva-Jothy 2011), and 38 μL of PBS, instead of 40 μL. The PO and proPO activities were expressed as the slope of the absorbance curve during its linear phase (Nicoletti et al. 2020).

#### Lytic Activity

To quantify lytic activity for each sample, 30 μL of each sample was added to 200 μL of a bacterial suspension (72 mg of lyophilized *Micrococcus lysodeikticus* in 20 mL PBS). Absorbance at 490 nm was recorded every 3 min for 60 min following a 15-min incubation at room temperature. Activity was calculated as the rate of OD reduction over time (Kortet et al. 2007).

#### Total Hemocyte Counts

Hemolymph was extracted from anesthetized worker bees by puncturing between abdominal segments III and IV (Chan et al. 2006). Approximately 5 µL per bee was collected in a glass capillary and transferred to a PCR tube containing 5 µL of trypan blue 0.4% in a 1:1 ratio. For total hemocyte counts, samples were transferred to dual chambers of a counting slide. Cell counts and viability were determined using the TC10 automated cell counter (Bio-Rad) following manufacturer protocols.

#### Protein Content

The total protein content in adults and pupa bees was determined using the Pierce BCA Protein Assay Kit (Thermo Scientific). For each sample, 5 μL was mixed with 45 μL of PBS and 150 μL of BCA reagent (A:B were placed in a radius of 50:1). Absorbance at 562 nm was measured after 15-min incubation at room temperature using a Multiskan GO Microplate reader (Thermo Scientific). The total protein content (μg/5 μL) was estimated using a standard BSA calibration curve.

### Statistical Analyses

Statistical analyses were performed in RStudio (version 2023.12.1 + 402). Chi-square tests were used to compare incoming vs. outgoing RFID records, behavioral categories, and daytime-scale frequencies, and *T*-tests were applied to compare activity duration between hives. Generalized linear models (GLMs) were used to analyze immune parameters. The unit of replication for protein and enzymatic assays (proPO, PO, lytic activity) was a pool of five bees, whereas hemocyte counts were obtained from individual bees. Because only two hives were studied, “Hive” (H1-healthy, H2-infested) was included as a fixed effect, with no random or blocking factors. Data transformations were applied where necessary according to model assumptions, in particular, proPO, PO, and lytic activity data were modeled with a Gamma distribution (link = log), and a constant of “1” was added to manage zeros in the dataset. We initially tested whether including “Age” (adults vs. pupae) or the interaction “Hive × Age” improved model fit, but models with “Hive” alone had the lowest AIC and were retained for parsimony. Parasite prevalence was analyzed by comparing the proportion of positive pools between the hives, and Levene’s test was used to assess variance homogeneity.

## Results

### Taxonomic and Molecular Classification of Phorid Flies

**Fig. 2:**
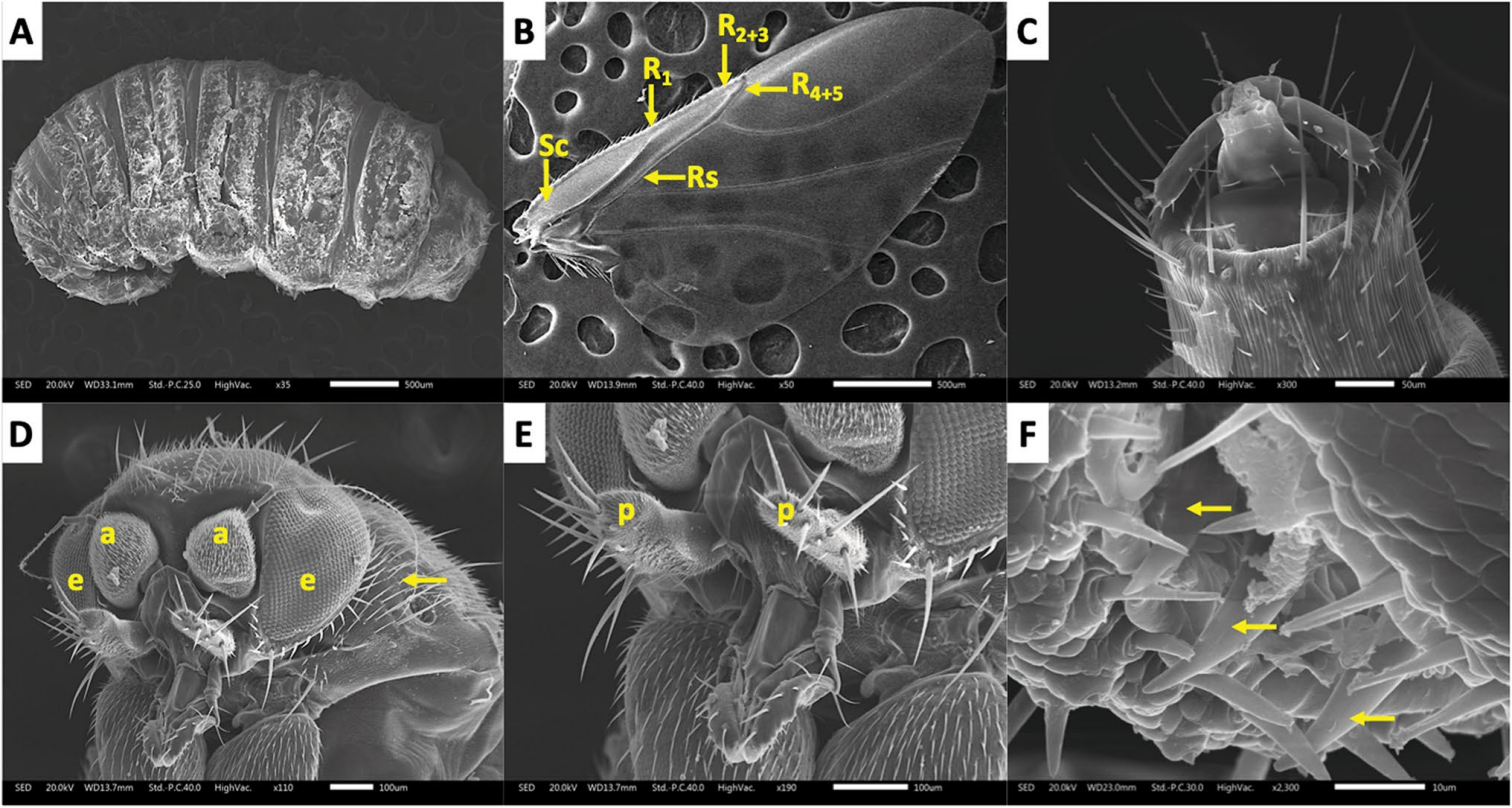
Morphological characteristics of *Megaselia* sp. by SEM micrographs. (**A**) Entire body view of a third-instar larva. (**B**) Wing venation highlighting radial veins (**R**), radial sector (Rs), and subcostal (Sc). (**C**) Apex of the female terminalia. (**D**): Propleura with numerous scattered hairs (yellow arrow), compound eyes (e), and antennae (a). (**E**) Maxillary palpi (p). (**F**) Mouthparts of *Megaselia* sp., ventrolateral view of labellum showing large, sharply pointed teeth (yellow arrows)

Adult phorid flies were classified based on morphological traits as outlined by Disney (1994, 2008). The specimens exhibited characteristic morphology of small flies (~ 2.5 mm), including a humpbacked thorax, laterally flattened hind femora, and distinctive wing venation indicative of the genus *Megaselia* (Fig. 2). Confirmation of their taxonomic classification was achieved through cytochrome oxidase I (COI) gene sequencing, which matched *Megaselia* sequences in the NCBI BLAST database (Online Resource 1).

### Observation of Parasitic Behavior in Honey Bee Hives

**Fig. 3:**
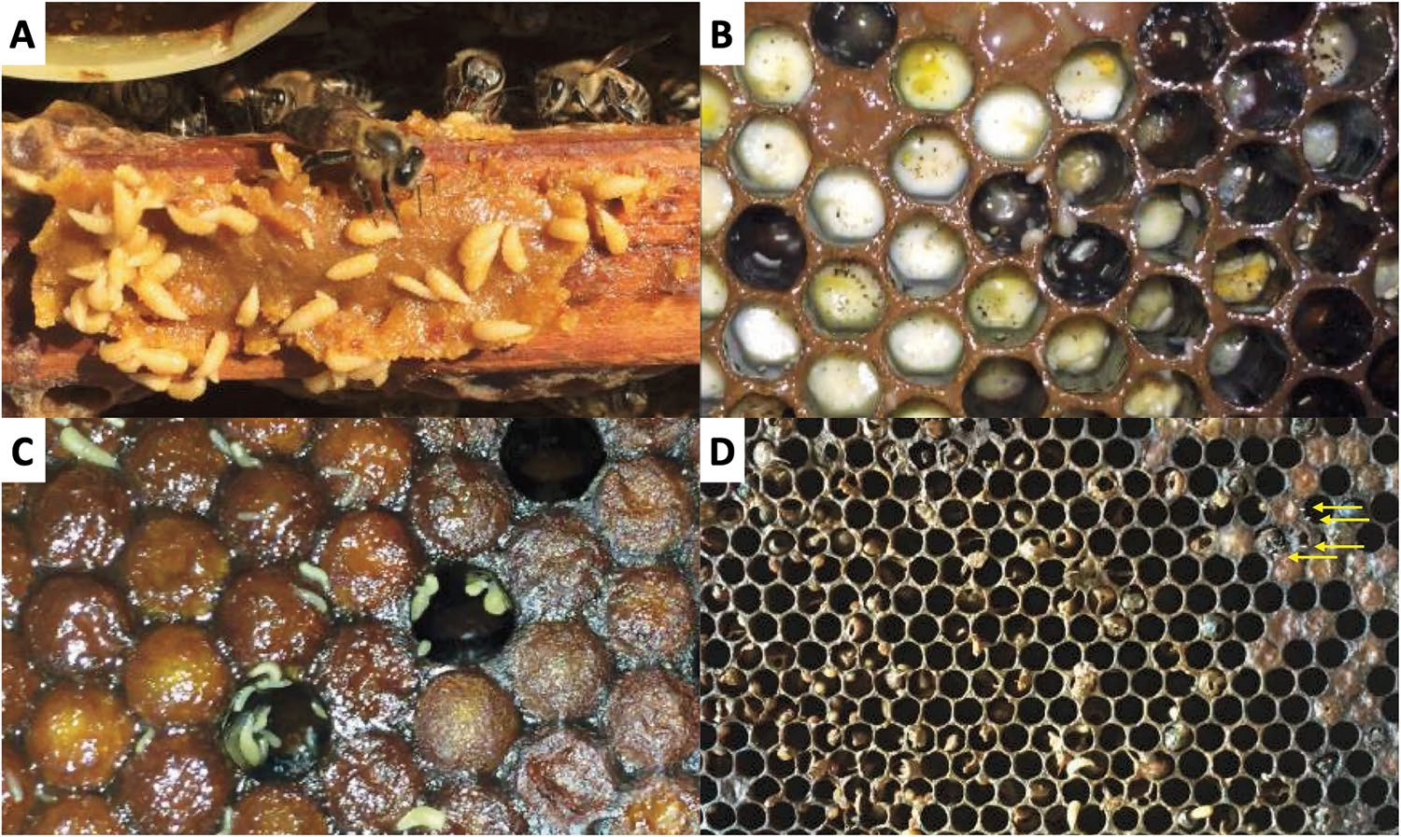
The H2-infested hive with *Megaselia* sp. eggs, larvae, and pupae. (**A**) Phorid fly larvae feeding on *Apis mellifera* food. (**B**) Phorid fly larvae of different ages feeding on *Apis mellifera* larvae. (**C**) Phorid fly larvae on capped brood cells. (**D**) Frame of the H2-infested hive showing the remains of *Apis mellifera* and *Megaselia* sp*.* after infestation (yellow arrows indicate some hatched eggs of phorid fly)

In the field, *Megaselia* larvae were observed on both adult bees and brood. Eggs, larvae, pupae, and adult phorids were observed in the brood frames of the H2-infested hive, whereas only larvae were observed on the hive’s stored food (Fig. 3). The densities of *Megaselia* were estimated at 318 larvae/10 cm^2^ and 291 pupae/10 cm^2^, averaging four pupae per cell. The eggs were rare on the adult bees and were laid mainly on capped and uncapped brood cells. The developmental stages observed suggest that the oviposition occurred approximately 3 weeks prior, while the bees were still alive. No phorid flies were detected in the H1-healthy hive.

### Bee Activity

**Fig. 4:**
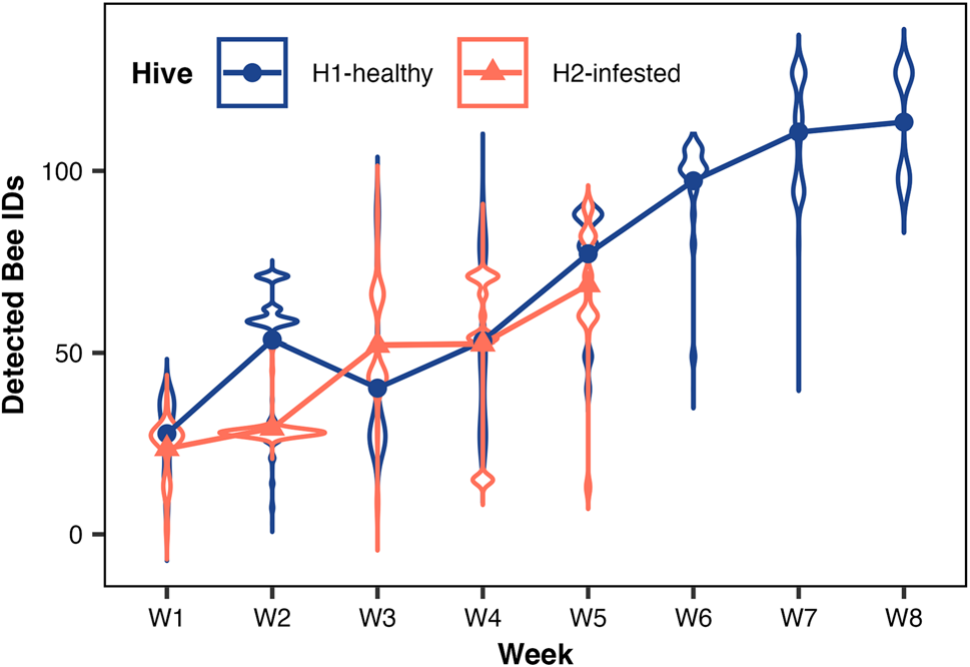
Violin plot with error bar shows the honey bee IDs detected weekly during monitoring. Both H1-healthy and H2-infested hives had a similar number of detected honey bees, except for W2 when the H1-healthy hive (blue circles) had a higher number of active honey bees than the H2-infested hive (orange triangles)

The bees of the H2-infested hive recorded a significantly higher number of incoming and outgoing RFID detections (*n* = 77,380; 93.1%) compared to the H1-healthy hive (*n* = 5762; 6.9%) (*χ*^2^ = 53,344, df = 7, *p* < 0.0001; Fig. 4, Table 3). Weekly comparisons showed similar numbers of detected bees in week 1 (W1) (*χ*^2^ = 2.79, df = 1, *p* = 0.09), W3 (*χ*^2^ = 3.31, df = 1, *p* = 0.07), W4 (*χ*^2^ = 0.29, df = 1, *p* = 0.59), and W5 (*χ*^2^ = 0.33, df = 1, *p* = 0.56). However, during W2, the H1-healthy hive displayed a significantly higher activity level than the H2-infested hive (*χ*^2^ = 10.13, df = 1, *p* = 0.001). By W5, the H2-infested hive had collapsed following the onset of *Megaselia* sp. oviposition in W2, leading to a subsequent surge in larval activity and pupation in W3. Post-collapse, the H1-healthy hive showed increasing bee activity, whereas the H2-infested hive recorded no further activity.

**Table 3:** Total filtered readings, weekly counts of RFID-detected honey bees, and behavioral category frequencies observed in H1-healthy and H2-infested hives

	H1-healthy	H2-infested	*χ*^2^	df	*p* value
Total filtered readings	5762	77,380	53,344	7	< 0.0001
Number of bees detected by week					
W1	10	19	2.79	1	0.09
W2	25	7	10.13	1	0.001
W3	19	32	3.31	1	0.07
W4	14	17	0.29	1	0.59
W5	5	7	0.33	1	0.56
W6	20	0	20.00	1	< 0.0001
W7	26	0	26.00	1	< 0.0001
W8	9	0	9.00	1	< 0.0001
Bees’ activity (behavior scale)					
BTE	4071	76,657	65,265	1	< 0.0001
SM	173	297	24.9	1	< 0.0001
FG	1433	373	622.2	1	< 0.0001
DB	85	53	7.4	1	0.006

Statistical results include Chi-square values, degrees of freedom, and *p* values

Bee behavior categories also differed between hives (*χ*^2^ = 16,454, df = 3, *p* < 0.0001). The H2-infested hive had more short activities “by the entry” (BTE; *χ*^2^ = 65,265, *p* < 0.0001) and “short mission” (SM; *χ*^2^ = 24.9, *p* < 0.0001) behaviors (76,657 and 297 detections for H2 *versus* 4071 and 173 detections for H1), while the H1-healthy hive showed more “foraging” (FG; *χ*^2^ = 622.2, *p* < 0.0001) and “departed bee” (DB; *χ*^2^ = 7.4, *p* = 0.006) behaviors (53 and 373 detections for H2-infested hive *versus* 85 and 1433 detections for H1-healthy hive, respectively) (Table 3). Importantly, when corrected by foraging effort (FG events), the probability of DB relative to FG was more than twice as high in the H2-infested hive (14.4%) compared with the H1-healthy hive (6.0%), indicating that bees from the infested colony were more likely to fail return from foraging.

**Fig. 5:**
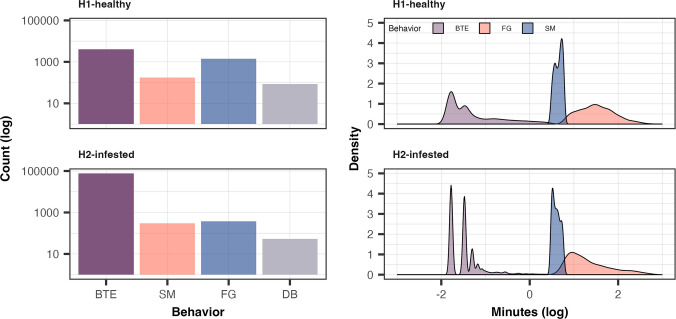
Behavioral activity over 8 weeks in the H1-healthy and H2-infested hives. The histogram shows the number of behaviors (log of count) and a density plot with each behavior’s duration (log of minutes): BTE, by the entry; DB, departed bee; FG, foraging; and SM, short mission. The H2-infested hive had more BTE and SM behaviors and fewer DB and FG behaviors than the H1-healthy hive. The BTE and FG behaviors had a shorter duration in the H2-infested hive than in the H1-healthy hive. DB behavior is excluded from the density plot as it represents unique detections without a duration. The log scale was required to display numerical data over a very wide range of values compactly

**Fig. 6:**
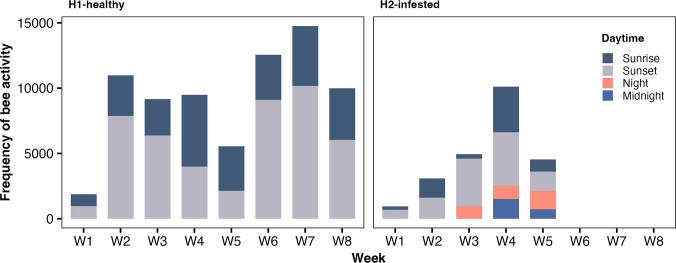
Frequency of bee activity at different daytimes for both hives. The H1-healthy hive was active at sunrise and sunset, while the H2-infested hive was active at sunrise, sunset, night, and midnight

There was also a significant difference in behavior duration between each category (*F* = 7.04, *p* < 0.0001). BTE and FG behaviors were significantly shorter in the H2-infested hive than in the H1-healthy hive (*p* < 0.0001, Fig. 5). Regarding the daily activity schedule, it was observed that the H1-healthy hive was active during the “sunrise” and “sunset” daytime categories, having a longer activity duration during the “sunset” (*t* = − 13.11, *p* < 0.0001). For hive H2-infested hive, two additional daytime categories were necessary, since it was also recorded during the “night” and “midnight.” At “midnight,” the activity duration was short (averaging 3.97 min) and was only recorded in W4 and W5, while the “night” included longer trips in W3 (*t* = − 35.54, *p* < 0.0001) and short trips in W4 (*t* = 3.73, *p* < 0.0001) and W5 (Fig. 6).

### Immune Response and Health

Molecular analysis by end-point PCR using specific diagnostic primer showed that adult worker bees and pupa pools from the H2-infested hive had a 0% prevalence of the *Ascosphaera apis*, *Melissococcus plutonius*, and *Vairimorpha ceranae*. In contrast, the H1-healthy showed no detection of *A. apis* and *M. plutonius* in pupae, but it presented a 100% prevalence of *V. ceranae* in adult worker bee pools, indicating that at least one individual per pool was infected (Table 2). Bees from H1-healthy and H2-infested hives had a similar protein content (GLM, *χ*^2^ = 1.0, *p* = 3.17), suggesting that the physiological condition of bees at the time of sampling was comparable, regardless of whether the colony was infested with phorid flies.

**Fig. 7:**
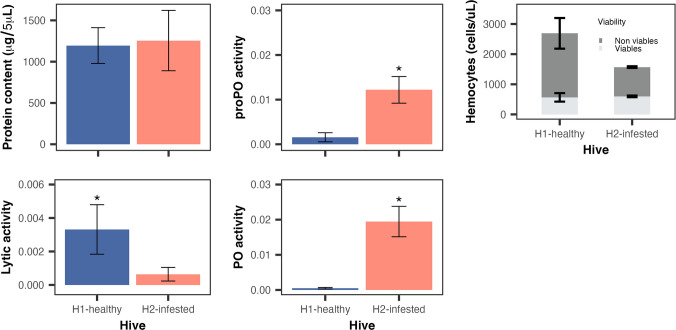
Humoral and cellular immunity of adult honey bees from H1-healthy and H2-infested hives. Protein content did not differ significantly between hives. However, the prophenoloxidase (proPO) and phenoloxidase (PO) activities were significantly higher in bees from the H2-infested hive, whereas lytic activity was significantly higher in bees from the H1-healthy hive. Total hemocyte counts were also higher in the H1-healthy hive, but the number of viable hemocytes did not differ between hives

Immune assays showed significantly higher proPO (GLM, *χ*^2^ = 13.98, *p* < 0.0001) and PO activity (GLM, *χ*^2^ = 22.05, *p* < 0.0001) in H2-infested hive compared to H1-healthy hive. However, lytic activity was higher in the H1-healthy hive (GLM, *χ*^2^ = 11.84, *p* < 0.001; Fig. 7). In all cases, the age of the bees did not significantly affect PO (*χ*^2^ = 0.008, *p* = 0.927), proPO (*χ*^2^ = 0.0001, *p* = 0.999), or lytic activity (*χ*^2^ = 1.71, *p* = 0.19). Total hemocyte counts (cells/mL) were significantly higher in the H1-healthy hive (*χ*^2^ = 145.22, *p* < 0.0001), whereas the number of viable hemocytes did not differ between hives (*χ*^2^ = 0.95, *p* = 0.33; Fig. 7).

## Discussion

Bees are generally more affected by macro- than microparasites. Boomsma et al. (2005) analyzed interactions between social hosts (bees, wasps, ants, and termites) and ten different parasites and diseases, showing that, after mites, dipterans are the most common. Phorid flies are a highly diverse dipteran group, often associated with social insects. Although their initial interactions are primarily detritivores or commensals (Hölldobler and Wilson 1990), increased population density or reduced food availability can trigger parasitic behavior (Wojcik 1994, Robroek et al. 2003). Several recent reports have documented the presence of phorid flies in *A. mellifera* colonies (Table 1). However, the physiological and behavioral impacts of these parasites on honey bees remain largely unexplored and are often underestimated. Although Cham et al. (2018) and Ricchiuti et al. (2016) provided preliminary evidence of *Megaselia* infestations in honey bees, no detailed field studies have evaluated their effects on bee behavior and defense mechanisms. Like other phorid species, *Megaselia* larvae can feed on bee tissues and hemolymph, potentially reducing the longevity of bees and impairing colony performance (Abou-Shaara and Darwish 2021). Our findings provide the first detailed description of this interaction, suggesting that phorid fly invasion may alter bee activity patterns and compromise colony condition.

The H2-infested hive showed increased short-duration activities (BTE and SM), shorter foraging trips (FG), and fewer departed bee events (DB) compared to the H1-healthy hive, possibly reflecting increased hive cleaning activity and short trips for essential functions such as excretion, resource exploration, and feeding in nearby points (Susanto et al. 2018). In addition, a decrease in honey bees may have altered the caste structure within the H2-infested hive. In this case, foraging bees, which normally make long trips in search of food, may have been forced to remain and defend the colony. A similar phenomenon was found by Mehdiabadi and Gilbert (2002) in the interaction between *A. mellifera* and the phorid fly *Pseudacteon tricuspis*, which decreased in half the worker population and reduced foraging rates for the social ant *Solenopsis invicta* (fire ants). Feener and Brown (1992) similarly observed that *Pseudacteon* spp*.* reduced foraging rates by up to 50% in the ant *Solenopsis geminata*. Bragança et al. (1998) also showed that relatively few leafcutter ants of *Atta sexdens* were attacked by the phorid fly *Neodohrniphora* sp., but these flies affected the behaviour of the outgoing foraging ants by making them defend the colony instead, this interaction caused a significant reduction in the number of foragers.

The *Megaselia* sp*.* invasion also appeared to affect the daily activity. H1-healthy hive had activityfrom sunrise to sunset, while the H2-infested hive was active at sunrise, sunset, night, and midnight. A similar behavior has been observed in the ant *Pheidole titanis*; the foraging workers stop foraging during the day because they defended the colony from the nocturnal oviposition attacks of parasitic phorid flies (Feener 1988). In this case, ants foraged at night when flies did not attacked them and took turns in their activity times to avoid parasitoids (Feener 1988). Core et al. (2012) associated the nocturnal activity of bees infested by phorids with hive abandonment, reporting that bees leaving the hive at night had higher parasitism than those leaving by day. They proposed that *A. borealis* could have manipulated the bees to the point of changing their circadian rhythms.

In our study, nocturnal activity in the H2-infested hive occurred mainly in weeks 4 and 5, with some evidence as early as week 3, during the period preceding colony collapse. The activity at “midnight” was short and restricted to weeks 4 and 5, while “night” activity included longer trips in week 3 and short trips in weeks 4 and 5. These events mostly consisted of short missions averaging 3.97 min, more consistent with host-driven stress or defensive responses (e.g., cleaning, guarding, agitation) than with sustained nocturnal foraging. This interpretation aligns with reports of nocturnal oviposition in *Megaselia* (Zulaikha and Zuha 2016), which may have triggered defensive activity, and with the strong hygienic behavior of Africanized bees (Aumeier et al. 2000), which often prioritize removal of infested individuals, eggs, or pupae at night. Such behaviors could explain repeated RFID detections near the reader as workers cleaned or defended the hive. At the same time, the nocturnal activity over several weeks does not rule out parasite-driven disruption of circadian rhythms. True manipulation should be expressed consistently across colonies and confer direct benefit to the parasite (Poulin 1995; Moore 2002), conditions not yet demonstrated for *Megaselia*. In our dataset, the proportion of “DB” (last detection) events relative to foraging trips “FG” was more than twice as high in H2-infested hive (14.4%) compared with H1-healthy hive (6.0%), suggesting that bees in the H2-infested hive were more likely to fail return, which could reflect stress-related losses or parasite induce effects. Future studies combining experimental infestations, dissections, or molecular screening of night active bees, and hygienic behavior assays will be necessary to determine whether nocturnal activity reflects host defenses or parasite manipulation or both. Alternative explanations such as RFID detection of dead bees removed by workers, or climatic effects, appear unlikely; meteorological data (Servicio Meteorológico Nacional (SMN), Comisión Nacional del Agua (CONAGUA) 2023) indicated stable conditions (9–26 °C) during the monitoring period.

Regarding the number of bees detected daily, both hives recorded similar bee IDs per week, except in W2, when fewer detected IDs were found in the H2-infested hive. This coincided with the estimated oviposition period of phorid flies in this hive. The lower number of detected IDs in W2 may reflect bees remaining inside the hive, as they spent their time cleaning cells and removing dead bodies and eggs, instead of foraging. The H2-infested hive may also have had weaker bees or a lower density, which could have favored the invasion and subsequent collapse.

Total protein concentration was similar between hives. Both hives were managed under identical conditions and had access to the same food sources. Given that detected IDs were similar between the two hives in the W1, for this reason, it is unlikely that the initial colony condition or the population density could predispose H2-infested hive to collapse, nor did it influence the bees’ ability to respond to the invasion or mount a defense against parasitoids. Although phorid flies are sometimes found on bees already dead, dying, or affected by other diseases (Dutto and Ferrazzi 2014), in our study, coinfections did not appear to predispose the H2-infested hive to collapse, with a zero prevalence of three of the most common parasites, *M. plutonius*, *A. apis*, and *V. ceranae*, which causes the European foulbrood, chalkbrood, and nosemosis diseases, respectively.

The strong proPO/PO activation observed in the H2-infested hive is consistent with defenses triggered by parasitoid oviposition, larval activity, and associated tissue damage (Pennacchio and Strand 2006). Although the ability of *A. mellifera* to melanize dipteran parasitoid eggs remains unclear, *A. mellifera* possesses a single phenoloxidase (AmPPO, Lourenço et al. 2005) which is activated through the proPO → PO cascade, typically induced against macroparasites such as nematodes or parasitoid eggs and larvae, although it can also act against some microparasites like bacteria or fungi. Our results suggest that enhanced PO activity reflects rapid activation of pre-existing proPO stores, allowing an immediate defense upon infection (Chan et al. 2009). In our assays, proPO and PO activities were measured as ΔAbs_490_/min in slope-based assays over 30 min, rather than *Vmax*, which typically yields higher absolute values (Wilson-Rich et al. 2008). In our data, proPO:PO ratios were close to 1 in both hives (H1-healthy hive: median 0.7, H2-infested hive: median 1.02), whereas Laughton et al. (2011) reported much lower ratios (~ 1:365 to ~ 1:50) in healthy bees, reflecting the predominance of inactive proPO in circulation. The ratios observed here suggest that a substantial fraction of the proPO pool had been converted into active PO, particularly in the H2-infested hive, consistent with a sustained immune activation. Melanization generally encapsulates large intruders, whereas against microparasites melanin tends to be more diffuse and acts through reactive intermediates (such as quinones and reactive radicals) with antimicrobial activity (Cerenius and Söderhäll 2004). Thus, the specific response of the honey bee immune system to *Megaselia* spp. oviposition remains an open question for further research.

In the H1-healthy hive, we detected a 100% prevalence of *V. ceranae*, but this was evaluated as presence/absence by endpoint PCR in pools. A positive pool means that at least one honey bee carried the parasite, but it does not indicate infection intensity. This point is important because parasite load (not measured here) could modulate both immune and behavioral responses. The higher lytic activity and hemocyte counts in the H1-healthy hive may reflect an antimicrobial response against *V. ceranae* since lysozymes and antimicrobial peptides are activated by Toll and IMD pathways to degrade the cellular layer of bacteria, fungi, and microsporidia (Keeling and Fast 2002; Lemaitre and Hoffmann 2007). Lytic activity is relatively stable under nutritional stress in other insects, even when other immune parameters vary (Adamo et al. 2016), supporting the idea that this pathway provides a robust line of defense. At high parasite loads, sustained lytic activity and cellular defense (granulocytes in larvae or plasmatocytes in adults) would be expected, although *V. ceranae* can suppress antimicrobial gene expression (suggesting that the microsporidium interferes with activation) and impair longevity and foraging (Antúnez et al. 2009; Higes et al. 2008). At low parasite loads, infections may appear with undetectable symptoms with limited impact on colony dynamics (Forsgren and Fries 2010), and in some cases even upregulate antimicrobial peptides (Lourenço et al. 2021). In fact, infected bees with *V. ceranae* have been shown to exhibit changes in their cuticular hydrocarbon (CHC) profiles but no significant differences in behavior or social interactions compared with healthy bees (McDonnell et al. 2013). We may speculate that the second scenario appears more consistent with our observations. Interestingly, although total hemocyte counts were significantly higher in the H1-healthy hive, the number of viable hemocytes did not differ between hives (H1, 566 cells/µL vs. H2, 599 cells/µL, see Fig. 7). Reported hemocyte concentrations in healthy honey bees average around 400–600 cells/µL (Wilson-Rich et al. 2008), which is comparable to the viable counts observed in both colonies. This suggests that the excess cells in H1-healthy hive consisted largely of non-viable hemocytes, possibly plasmatocytes undergoing rupture or apoptosis during melanization or coagulation (Terán-Murillo et al. 2025). Similar processes have been documented in *Drosophila* and Lepidoptera, where melanotic encapsulation involves hemocyte rupture and apoptotic pathways (Eleftherianos et al. 2021). Hemocyte death is recognized as a biologically relevant outcome of immune activation (Terán-Murillo et al. 2025). Hemocyte dynamics are also shaped by intrinsic and extrinsic factors; for example, counts decline with age but not task (Wilson-Rich et al. 2008), and experimental infections with *Serratia marcescens* reduce hemocyte numbers compared with asymptomatic bees (Burritt et al. 2016). In our study, the elevated but largely non-viable hemocyte count in the H1-healthy hive is consistent with immune activation and turnover under *V. ceranae infection*, in contrast to the reduced hemocytes in the H2-infested hive under phorid fly pressure.

In addition, trade-offs between lytic activity and proPO/PO pathways have been documented (Cotter et al. 2004; Schmid-Hempel 2005; Chan et al. 2009; Nicoletti et al. 2020). Our results support this trade-off because investment in lytic activity in the H1-healthy hive could have limited proPO/PO activation, whereas the opposite pattern prevailed in the H2-infested hive. However, there is no evidence of a trade-off between lytic activity and hemocyte counts in the H1-healthy hive. Moreover, the lower hemocyte counts in the H2-infested hive could reflect resource depletion or immune exhaustion after sustained activation of the proPO/PO pathway, as hemocytes are required both for both melanization and wound repair. Such patterns may reflect immune system reconfiguration rather than simple trade-offs (Adamo 2017), where some pathways (e.g., lytic activity) are maintained as robust front-line defenses while others (e.g., proPO/PO cascade) are differentially modulated depending on the stressor.

The coinfections in social insects are not yet well understood. Core et al. (2012) found that *V. ceranae* had a prevalence of 50% in adult phorids and 87.7% in larvae, while the deformed wing virus (DWV) had a prevalence of 25% in adult phorids and 75% in larvae. This suggests that phorids may selectively avoid hosts already harboring infections, possibly by detecting volatiles from infected bees, selecting hives free of other parasites before invading them. Richard et al. (2008) showed that experimentally infected bees with lipopolysaccharides significantly expressed different cuticular hydrocarbon profiles compared to healthy bees, emitting specific volatiles that other parasites might detect. In our case, although both hives were adjacent, it is possible that H1-healthy hive, already infected with *V. ceranae*, was less attractive, whereas phorids invaded the parasite-free H2-infested hive as a higher-quality resource to develop their offspring.

It is important to note that this study was based on the comparison of a single infested hive and a single healthy hive. Consequently, our results should be regarded as a case study rather than a generalized pattern. Although the behavioral and immunological changes we observed are consistent with beekeeper reports of colony abandonment and collapse following phorid infestations, replicated studies including more colonies and sites will be necessary to confirm these trends and to clarify the mechanisms involved.

Phorid species can be considered an emerging threat that may also affect native bees (Core et al. 2012) that could contribute to colony weakening and finally collapse or abandonment, reinforcing the need for improved management strategies. More attention should be given to a potential increase of infestations by phorid flies in honey bee colonies. For beekeepers, early detection of parasites is crucial for maintaining a healthy and productive apiary, and the infected hives are constantly monitored and treated until collapse or clearance; however, the healthy hives are monitored quickly or less frequently.

Future research should test whether the behavioral and immunological patterns reported here are consistent across multiple colonies and landscapes. A key avenue will be to evaluate the hypothesis that phorids might selectively target uninfected or healthier hives (without or with a low prevalence of microparasites), possibly because they represent higher-quality resources for fly larval development. Therefore, we emphasize the importance of giving special attention to the management of healthy hives and not taking for granted their good condition over time. Confirming or rejecting this idea under replicated and controlled conditions will be essential to clarify the dynamics of phorid infestations and their consequences for honey bee health.

## Supplementary Information

Below is the link to the electronic supplementary material.

Supplementary file1 (DOCX 2.27 MB)

## Data Availability

Data will be available from the corresponding author on reasonable request.

## References

[CR1] Abou-Shaara HF, Darwish AA (2021) Expected prevalence of the facultative parasitoid *Megaselia scalaris* of honey bees in Africa and the Mediterranean region under climate change conditions. Int J Trop Insect Sci. 10.1007/s42690-021-00508-5

[CR2] Adamo SA (2017) Stress responses sculpt the insect immune system, optimizing defense in an ever-changing world. Dev Comp Immunol 66:24–32. 10.1016/j.dci.2016.06.005 27288849 10.1016/j.dci.2016.06.005

[CR3] Adamo SA, Davies G, Easy R, Kovalko I, Turnbull KF (2016) Reconfiguration of the immune system network during food limitation in the caterpillar *Manduca sexta*. J Exp Biol 219(5):706–718. 10.1242/jeb.132936 26747906 10.1242/jeb.132936

[CR4] Antúnez K, Martín-Hernández R, Prieto L, Meana A, Zunino P, Higes M (2009) Immune suppression in the honey bee (*Apis mellifera*) following infection by *Nosema ceranae*. Environ Microbiol 11(9):2284–2290. 10.1111/j.1462-2920.2009.01953.x 19737304 10.1111/j.1462-2920.2009.01953.x

[CR5] Ashworth L, Quesada M, Casas A, Aguilar R, Oyama K (2009) Pollinator-dependent food production in Mexico. Biol Conserv 142(5):1050–1057. 10.1016/j.biocon.2009.01.016

[CR6] Aumeier P, Rosenkranz P, Gonçalves LS (2000) A comparison of the hygienic response of Africanized and European (*Apis mellifera carnica*) honey bees to *Varroa*-infested brood in tropical Brazil. Genet Mol Biol 23:787–791. 10.1590/S1415-47572000000400013

[CR7] Ayup MM, Gaertner P, Agosto-Rivera JL, Marendy P, de Souza P, Galindo-Cardona A (2021) Analysis of honeybee drone activity during the mating season in Northwestern Argentina. Insects 12(6):566. 10.3390/insects12060566 34205532 10.3390/insects12060566 PMC8234112

[CR8] Balvino-Olvera FJ, Olivares-Pinto U, González-Rodríguez A, Aguilar-Aguilar MJ, Ruiz-Guzmán G, Lobo-Segura J, Quesada M (2024) Effects of floral resources on honey bee populations in Mexico: using dietary metabarcoding to examine landscape quality in agroecosystems. Ecol Evol 14(6):e11456. 10.1002/ece3.11456 38895569 10.1002/ece3.11456 PMC11183941

[CR9] Boehme P, Amendt J, Disney RHL, Zehner R (2010) Molecular identification of carrion-breeding scuttle flies (Diptera: Phoridae) using COI barcodes. Int J Legal Med 124(6):577–581. 10.1007/s00414-010-0429-5 20195623 10.1007/s00414-010-0429-5

[CR10] Boomsma JJ, Schmid-Hempel P, Hughes WOH (2005) Life histories and parasite pressure across the major groups of social insects. In: Fellowes MDE, Holloway GJ, Rolff J (eds) Insect evolutionary ecology: proceedings of the royal entomological society’s 22nd symposium, reading, UK, 2003. CABI Publishing, Wallingford, pp 139–175

[CR11] Bragança MAL, Tonhasca A Jr., Della Lucia TM (1998) Reduction in the foraging activity of the leaf-cutting ant *Atta sexdens* caused by the phorid *Neodohrniphora sp*. Entomol Exp Appl 89(3):305–311. 10.1046/j.1570-7458.1998.00413.x

[CR12] Brodschneider R, Gray A, Adjlane N, Ballis A, Brusbardis V, Charrière JD, Danihlík J et al (2018) Multi-country loss rates of honey bee colonies during winter 2016/2017 from the COLOSS survey. J Apic Res 57(3):452–457. 10.1080/00218839.2018.1460911

[CR13] Burciaga RA, Ruiz-Guzmán G, Lanz-Mendoza H, Krams I, Contreras-Garduño J (2023) The honey bees immune memory. Dev Comp Immunol 138:104528. 10.1016/j.dci.2022.104528 36067906 10.1016/j.dci.2022.104528

[CR14] Burritt NL, Foss NJ, Neeno-Eckwall EC, Church JO, Hilger AM, Hildebrand JA, Warshauer DM, Perna NT, Burritt JB (2016) Sepsis and hemocyte changes in honey bees (*Apis mellifera*) experimentally infected with *Serratia marcescens*. PLoS ONE 11(9):e0162752. 10.1371/journal.pone.0167752 28002470 10.1371/journal.pone.0167752 PMC5176276

[CR15] Cerenius L, Söderhäll K (2004) The prophenoloxidase-activating system in invertebrates. Immunol Rev 198(1):116–126. 10.1111/j.0105-2896.2004.00116.x 15199959 10.1111/j.0105-2896.2004.00116.x

[CR16] Cham DT, Fombong AT, Ndegwa PN, Irungu LW, Nguku E, Raina SK (2018) *Megaselia scalaris* (Diptera: Phoridae), an opportunist parasitoid of honey bees in Cameroon. Afr Entomol 26(1):254–258. 10.4001/003.026.0254

[CR17] Chan QW, Howes CG, Foster LJ (2006) Quantitative comparison of caste differences in honeybee hemolymph* S. Mol Cell Proteom 5(12):2252–2262. 10.1074/mcp.M600197-MCP200 10.1074/mcp.M600197-MCP200 16920818

[CR18] Chan QW, Melathopoulos AP, Pernal SF, Foster LJ (2009) The innate immune and systemic response in honey bees to a bacterial pathogen. Paenibacillus Larvae BMC Genom 10(1):387–389. 10.1186/1471-2164-10-387 10.1186/1471-2164-10-387 PMC2907699 19695106

[CR19] Contreras-Garduño J, Lanz-Mendoza H, Córdoba-Aguilar A (2007) The expression of a sexually selected trait correlates with different immune defense components and survival in males of the *American rubyspot*. J Insect Physiol 53(6):612–621. 10.1016/j.jinsphys.2007.03.003 17451742 10.1016/j.jinsphys.2007.03.003

[CR20] Core A, Runckel C, Ivers J, Quock C, Siapno T, DeNault S, Hafernik J et al (2012) A new threat to honey bees, the parasitic phorid fly *Apocephalus borealis*. PLoS ONE 7(1):e29639. 10.1371/journal.pone.0029639 22235317 10.1371/journal.pone.0029639 PMC3250467

[CR21] Cotter SC, Kruuk LEB, Wilson K (2004) Costs of resistance: genetic correlations and potential trade-offs in an insect immune system. J Evol Biol 17(2):421–429. 10.1046/j.1420-9101.2003.00655.x 15009275 10.1046/j.1420-9101.2003.00655.x

[CR22] Cremer S (2019) Social immunity in insects. Curr Biol 29(11):R458–R463. 10.1016/j.cub.2019.03.035 31163158 10.1016/j.cub.2019.03.035

[CR23] Currie RW, Pernal SF, Guzmán-Novoa E (2010) Honey bee colony losses in Canada. J Apic Res 49(1):104–106. 10.3896/IBRA.1.49.1.18

[CR24] de Souza P, Marendy P, Barbosa K, Budi S, Hirsch P, Nikolic N, Gunthorpe T, Pessin G, Davie A (2018) Low-cost electronic tagging system for bee monitoring. Sensors 18:2124. 10.3390/s18072124 30004457 10.3390/s18072124 PMC6068632

[CR25] Dias de Freitas C, Oki Y, Resende FM, Zamudio F, Simone de Freitas G, Moreira de Rezende K et al (2023) Impacts of pests and diseases on the decline of managed bees in Brazil: a beekeeper perspective. J Apic Res 62(5):969–982. 10.1080/00218839.2022.2099188

[CR26] Disney RHL (2008) Natural history of the scuttle fly, *Megaselia scalaris*. Annu Rev Entomol 53:39–60. 10.1146/annurev.ento.53.103106.093415 17622197 10.1146/annurev.ento.53.103106.093415

[CR27] Disney RHL (1994) Scuttle flies: the Phoridae. Chapman & Hall. 10.1007/978-94-011-1288-8

[CR28] Doyle J (1991) DNA protocols for plants. In: Molecular techniques in taxonomy. Springer, Berlin, Heidelberg pp. 283–293. 10.1007/978-3-642-83962-7_18

[CR29] Dutto M, Ferrazzi P (2014) *Megaselia rufipes* (Diptera: Phoridae): a new cause of facultative parasitoidism in *Apis mellifera*. J Apic Res 53(1):141–145. 10.3896/IBRA.1.53.1.15

[CR30] Eleftherianos I, Baldwin H, Reynolds SE (2008) Developmental modulation of immunity: changes within the feeding period of the fifth larval stage in the defence reactions of *Manduca sexta* to infection by *Photorhabdus*. J Insect Physiol 54(1):309–318. 10.1016/j.jinsphys.2007.10.003 18001766 10.1016/j.jinsphys.2007.10.003

[CR31] Eleftherianos I, Heryanto C, Bassal T, Zhang W, Tettamanti G, Mohamed A (2021) Haemocyte-mediated immunity in insects: cells, processes and associated components in the fight against pathogens and parasites. Immunol 164(3):401–432. 10.1111/imm.13390 10.1111/imm.13390 PMC8517599 34233014

[CR32] Feener DH (1988) Effects of parasites on foraging and defense behavior of a termitophagous ant, *Pheidole titanis* Wheeler (Hymenoptera: Formicidae). Behav Ecol Sociobiol 22(6):421–427. 10.1007/BF00294980

[CR33] Feener DH Jr, Brown BV (1992) Reduced foraging of Solenopsis geminata (Hymenoptera: Formicidae) in the presence of parasitic Pseudacteon spp. (Diptera: Phoridae). Annu Entomol Soc Am 85(1):80–84. 10.1093/aesa/85.1.80

[CR34] Folmer O, Black M, Hoeh W, Lutz R, Vrijenhoek R (1994) DNA primers for amplification of mitochondrial cytochrome c oxidase subunit I from diverse metazoan invertebrates. Mol Mar Biol Biotechnol 3:294–299 7881515

[CR35] Forsgren E, Fries I (2010) Comparative virulence of *Nosema ceranae* and *Nosema apis* in individual European honey bees. Vet Parasitol 170(3–4):212–217. 10.1016/j.vetpar.2010.02.010 20299152 10.1016/j.vetpar.2010.02.010

[CR36] Garrido-Bailón E, Higes M, Martínez-Salvador A, Antúnez K, Botías C, Meana A, Martín-Hernández R et al (2013) The prevalence of the honeybee brood pathogens *Ascosphaera apis*, *Paenibacillus larvae* and *Melissococcus plutonius* in Spanish apiaries determined with a new multiplex PCR assay. Microb Biotechnol 6(6):731–739. 10.1111/1751-7915.12070 23919248 10.1111/1751-7915.12070 PMC3815939

[CR37] Gillespie JP, Kanost MR, Trenczek T (1997) Biological mediators of insect immunity. Annu Rev Entomol 42:611–643. 10.1146/annurev.ento.42.1.611 9017902 10.1146/annurev.ento.42.1.611

[CR38] Gray A, Adjlane N, Arab A, Ballis A, Brusbardis V, Bugeja Douglas A et al (2023) Honey bee colony loss rates in 37 countries using the COLOSS survey for winter 2019–2020: the combined effects of operation size, migration and queen replacement. J Apic Res 62(2):204–210. 10.1080/00218839.2022.2113329

[CR39] Graystock P, Goulson D, Hughes WO (2015) Parasites in bloom: flowers aid dispersal and transmission of pollinator parasites within and between bee species. Proc R Soc Lond B Biol Sci 282(1813):20151371. 10.1098/rspb.2015.1371 10.1098/rspb.2015.1371 PMC4632632 26246556

[CR40] Higes M, Martín-Hernández R, Botías C, Bailón EG, González-Porto AV, Barrios L, Del Nozal MJ, Bernal JL, Jiménez JJ, Palencia PG, Meana A (2008) How natural infection by *Nosema ceranae* causes honeybee colony collapse. Environ Microbiol 10(10):2659–2669. 10.1111/j.1462-2920.2008.01687.x 18647336 10.1111/j.1462-2920.2008.01687.x

[CR41] Hill GE (2011) Condition-dependent traits as signals of the functionality of vital cellular processes. Ecol Lett 14(7):625–634. 10.1111/j.1461-0248.2011.01622.x 21518211 10.1111/j.1461-0248.2011.01622.x

[CR42] Hölldobler B, Wilson EO (1990) The ants. Belknap Press of Harvard University Press, Cambridge

[CR43] Hristov P, Shumkova R, Palova N, Neov B (2020) Factors associated with honey bee colony losses: a mini-review. Vet Sci 7(4):166. 10.3390/vetsci7040166 33143134 10.3390/vetsci7040166 PMC7712510

[CR44] Kanost MR, Jiang H, Yu XQ (2004) Innate immune responses of a lepidopteran insect, *Manduca sexta*. Immunol Rev 198:97–105. 10.1111/j.0105-2896.2004.0121.x 15199957 10.1111/j.0105-2896.2004.0121.x

[CR45] Keeling PJ, Fast NM (2002) Microsporidia: biology and evolution of highly reduced intracellular parasites. Annu Rev Microbiol 56:93–116. 10.1146/annurev.micro.56.012302.160854 12142484 10.1146/annurev.micro.56.012302.160854

[CR46] Khattab MM, El-Hosseny EN (2014) The first records of the parasite zombie fly (*Apocephalus borealis* Brues) on honeybee, *Apis mellifera* in Egypt. Int J Agric Sci Res 4(6):37–42

[CR47] Koch H, Brown MJ, Stevenson PC (2017) The role of disease in bee foraging ecology. Curr Opin Insect Sci 21:60–67. 10.1016/j.cois.2017.05.008 28822490 10.1016/j.cois.2017.05.008

[CR48] Kortet R, Rantala MJ, Hedrick A (2007) Boldness in anti-predator behaviour and immune defence in field crickets. Evol Ecol Res 9(1):185–197

[CR49] Lamas ZS, Chen Y, Evans JD (2024) Case report: emerging losses of managed honey bee colonies. Biology 13(2):117. 10.3390/biology13020117 38392335 10.3390/biology13020117 PMC10887003

[CR50] Laughton AM, Siva-Jothy MT (2011) A standardised protocol for measuring phenoloxidase and prophenoloxidase in the honey bee, Apis mellifera. Apidologie 42(2):140–149

[CR51] Laughton AM, Boots M, Siva-Jothy MT (2011) The ontogeny of immunity in the honey bee, *Apis mellifera* L. following an immune challenge. J Insect Physiol 57(7):1023–1032. 10.1016/j.jinsphys.2011.04.020 21570403 10.1016/j.jinsphys.2011.04.020

[CR52] Lee KP, Simpson SJ, Wilson K (2008) Dietary protein-quality influences melanization and immune function in an insect. Funct Ecol 22(6):1052–1061. 10.1111/j.1365-2435.2008.01459.x

[CR53] Lemaitre B, Hoffmann J (2007) The host defense of *Drosophila melanogaster*. Annu Rev Immunol 25:697–743. 10.1146/annurev.immunol.25.022106.141615 17201680 10.1146/annurev.immunol.25.022106.141615

[CR54] Lourenço AP, Zufelato MS, Bitondi MMG, Simões ZLP (2005) Molecular characterization of a cDNA encoding prophenoloxidase and its expression in *Apis mellifera*. Insect Biochem Mol Biol 35(6):541–552. 10.1016/j.ibmb.2005.01.013 15857760 10.1016/j.ibmb.2005.01.013

[CR55] Lourenço AP, Guidugli-Lazzarini KR, de Freitas NH, Message D, Bitondi MM, Simões ZL, Teixeira ÉW (2021) Immunity and physiological changes in adult honey bees (*Apis mellifera*) infected with *Nosema ceranae*: The natural colony environment. J Insect Physiol 131:104237. 10.1016/j.jinsphys.2021.104237 33831437 10.1016/j.jinsphys.2021.104237

[CR56] Martín-Hernández R, Meana A, Prieto L, Salvador AM, Garrido-Bailón E, Higes M (2007) Outcome of colonization of *Apis mellifera* by *Nosema ceranae*. Appl Environ Microbiol 73(20):6331–6338. 10.1128/AEM.00270-07 17675417 10.1128/AEM.00270-07 PMC2075036

[CR57] McDonnell CM, Alaux C, Parrinello H, Desvignes JP, Crauser D, Durbesson E, ... Le Conte Y (2013) Ecto-and endoparasite induce similar chemical and brain neurogenomic responses in the honey bee (*Apis mellifera*). BMC Ecol 13(1):25. 10.1186/1472-6785-13-25 10.1186/1472-6785-13-25 PMC3725162 23866001

[CR58] Medina-Flores CA, López-Carlos M, Carrillo-Muro O, Gray A (2023) Honey bee colony losses in Mexico’s semi-arid high plateau for the winters 2016–2017 to 2021–2022. Insects 14(5):453. 10.3390/insects14050453 37233081 10.3390/insects14050453 PMC10231095

[CR59] Mehdiabadi NJ, Gilbert LE (2002) Colony–level impacts of parasitoid flies on fire ants. Proc R Soc Lond B Biol Sci 269(1501):1695–1699. 10.1098/rspb.2002.2087 10.1098/rspb.2002.2087 PMC1691088 12204130

[CR60] Menail AH, Piot N, Meeus I, Smagghe G, Loucif-Ayad W (2016) Large pathogen screening reveals first report of *Megaselia scalaris* (Diptera: Phoridae) parasitizing *Apis mellifera intermissa* (Hymenoptera: Apidae). J Invertebr Pathol 137:33–37. 10.1016/j.jip.2016.04.007 27130035 10.1016/j.jip.2016.04.007

[CR61] Mohammed SEAR (2018) First report of *Apis mellifera carnica* Ruttner (Hymenoptera, Apidae) in Saudi Arabia parasitized by a phorid parasitoid (Diptera: Phoridae). J Apic Res 57(4):565–568. 10.1080/00218839.2018.1466760

[CR62] Moore J (2002) Parasites and the behavior of animals. Oxford University Press, New York.

[CR63] Nicoletti M, Gilles F, Galicia-Mendoza I, Rendón-Salinas E, Alonso A, Contreras-Garduño J (2020) Physiological costs in monarch butterflies due to forest cover and visitors. Ecol Indic 117:106592. 10.1016/j.ecolind.2020.106592

[CR64] Novais SM, Nunes CA, Santos NB, DAmico AR, Fernandes GW, Quesada M, Neves ACO et al (2016) Effects of a possible pollinator crisis on food crop production in Brazil. PLoS ONE 11(11):e0167292. 10.1371/journal.pone.0167292 27902787 10.1371/journal.pone.0167292 PMC5130262

[CR65] Nunes-Silva P, Hrncir M, Guimarães JTF, Arruda H, Costa L, Pessin G, Imperatriz-Fonseca VL et al (2019) Applications of RFID technology on the study of bees. Insectes Soc 66(1):15–24. 10.1007/s00040-018-0660-5

[CR66] Oliveira RC, Contrera FAL, Arruda H, Jaffe R, Pessin G, Venturieri GC, de Souza P, Imperatriz-Fonseca VL (2021) Foraging and drifting patterns of a highly eusocial neotropical stingless bee species assessed by radio-frequency identification tags. Front Ecol Evol 9:708178. 10.3389/fevo.2021.708178

[CR67] Pennacchio F, Strand MR (2006) Evolution of developmental strategies in parasitic Hymenoptera. Annu Rev Entomol 51:233–258. 10.1146/annurev.ento.51.110104.151029 16332211 10.1146/annurev.ento.51.110104.151029

[CR68] Perry CJ, Søvik E, Myerscough MR, Barron AB (2015) Rapid behavioral maturation accelerates failure of stressed honey bee colonies. Proc Natl Acad Sci 112(11):3427–3432. 10.1073/pnas.1422089112 25675508 10.1073/pnas.1422089112 PMC4371971

[CR69] Polatto LP, Chaud-Netto J, Alves-Junior VV (2014) Influence of abiotic factors and floral resource availability on daily foraging activity of bees. J Insect Behav 27(5):593–612. 10.1007/s10905-014-9452-6

[CR70] Potts SG, Imperatriz-Fonseca V, Ngo HT, Aizen MA, Biesmeijer JC, Breeze TD, Vanbergen AJ et al (2016) Safeguarding pollinators and their values to human well-being. Nature 540(7632):220–229. 10.1038/nature20588 27894123 10.1038/nature20588

[CR71] Poulin R (1995) Adaptive changes in host behaviour by parasites: a review. Int J Parasitol 25(12):1371–1383. 10.1016/0020-7519(95)00100-X 8719948 10.1016/0020-7519(95)00100-x

[CR72] Ratcliffe NA, Leonard C, Rowley AF (1984) Prophenoloxidase activation: nonself recognition and cell cooperation in insect immunity. Science 226(4674):557–559. 10.1126/science.226.4674.557 17821514 10.1126/science.226.4674.557

[CR73] Requier F, Leyton MS, Morales CL, Garibaldi LA, Giacobino A, Porrini MP et al (2024) First large-scale study reveals important losses of managed honey bee and stingless bee colonies in Latin America. Sci Rep 14(1):10079. 10.1038/s41598-024-59513-6 38698037 10.1038/s41598-024-59513-6 PMC11066017

[CR74] Ricchiuti L, Miranda M, Venti R, Bosi F, Marino L, Mutinelli F (2016) Infestation of *Apis mellifera* colonies by *Megaselia scalaris* (Loew, 1866) in Abruzzo and Molise regions, central-southern Italy. J Apic Res 55(2):187–192. 10.1080/00218839.2016.1196017

[CR75] Richard FJ, Aubert A, Grozinger CM (2008) Modulation of social interactions by immune stimulation in honey bee, *Apis mellifera*, workers. BMC Biol 6(1):1–13. 10.1186/1741-7007-6-50 19014614 10.1186/1741-7007-6-50 PMC2596086

[CR76] Robroek BJ, de Jong H, Sommeijer MJ (2003) The behaviour of the kleptoparasite, *Pseudohypocera kerteszi* (Diptera, Phoridae), in hives of stingless bees (Hymenoptera, Apidae) in Central America. In: Proceedings Of The Section Experimental And Applied Entomology-Netherlands Entomological Society, pp 65–70

[CR77] Roy D, Debnath P, Mondal D, Sarkar P (2018) Colony collapse disorder of honey bee: a neoteric ruction in global apiculture. Curr J Appl Sci Technol 26(3):1–12. 10.9734/CJAST/2018/38218

[CR78] Sabo R, Legáth J, Staroň M, Sabová L (2020) The first record of facultative parasitism of spp (Diptera:). in a honeybee colony in Slovakia. Folia Vet 64(4):44–48. 10.2478/fv-2020-0036

[CR79] Schmid-Hempel P (2005) Evolutionary ecology of insect immune defenses. Annu Rev Entomol 50:529–551. 10.1146/annurev.ento.50.071803.130420 15471530 10.1146/annurev.ento.50.071803.130420

[CR80] Servicio Meteorológico Nacional (SMN), Comisión Nacional del Agua (CONAGUA) (2023) Información estadística climatológica. Available at: https://smn.conagua.gob.mx/es/climatologia/informacion-climatologica/informacion-estadistica-climatologica . Accessed 14 Aug 2023

[CR81] Simmons BI, Balmford A, Bladon AJ, Christie AP, De Palma A, Dicks LV et al (2019) Worldwide insect declines: an important message, but interpret with caution. Ecol Evol 9(7):3678–3680. 10.1002/ece3.5153 31015957 10.1002/ece3.5153 PMC6467851

[CR82] Söderhäll K, Cerenius L (1998) Role of the prophenoloxidase-activating system in invertebrate immunity. Curr Opin Immunol 10(1):23–28. 10.1016/S0952-7915(98)80026-5 9523106 10.1016/s0952-7915(98)80026-5

[CR83] Susanto F, Gillard T, De Souza P, Vincent B, Budi S, Almeida A, He J (2018) Addressing RFID misreadings to better infer bee hive activity. IEEE Access 6:31935–31949. 10.1109/ACCESS.2018.2844181

[CR84] Tang H, Kambris Z, Lemaitre B, Hashimoto C (2006) Two proteases defining a melanization cascade in the immune system of *Drosophila*. J Biol Chem 281(38):28097–28104. 10.1074/jbc.M601642200 16861233 10.1074/jbc.M601642200

[CR85] Terán-Murillo F, Ghosh E, Rantala MJ, Krams I, Krams R, Contreras-Garduño J (2025) Does immune priming in *Galleria mellonella* reveal plastic mechanisms for survival? Dev Comp Immunol. 10.1016/j.dci.2025.105407 40582433 10.1016/j.dci.2025.105407

[CR86] Toppa RH, Arena MVN, da Silva CI, Marendy P, de Souza P, da Silva-Zacarin ECM (2020) Impact of glues used for RFIDs on the longevity and flight muscle of the stingless bee *Melipona quadrifasciata* (Apidae: Meliponini). Apidologie 52:328–340. 10.1007/s13592-020-00823-9

[CR87] Truong AT, Yoo MS, Seo SK, Hwang TJ, Yoon SS, Cho YS (2023) Prevalence of honey bee pathogens and parasites in South Korea: a five-year surveillance study from 2017 to 2021. Heliyon. 10.1016/j.heliyon.2023.e13494 36816323 10.1016/j.heliyon.2023.e13494 PMC9929316

[CR88] vanEngelsdorp D, Evans JD, Saegerman C, Mullin C, Haubruge E, Nguyen BK, Pettis JS et al (2009) Colony collapse disorder: a descriptive study. PLoS ONE 4(8):e6481. 10.1371/journal.pone.0006481 19649264 10.1371/journal.pone.0006481 PMC2715894

[CR89] Wilson-Rich N, Dres ST, Starks PT (2008) The ontogeny of immunity: development of innate immune strength in the honey bee (*Apis mellifera*). J Insect Physiol 54(10–11):1392–1399. 10.1016/j.jinsphys.2008.07.016 18761014 10.1016/j.jinsphys.2008.07.016

[CR90] Wilson-Rich N, Spivak M, Fefferman NH, Starks PT (2009) Genetic, individual, and group facilitation of disease resistance in insect societies. Annu Rev Entomol 54:405–423. 10.1146/annurev.ento.53.103106.093301 18793100 10.1146/annurev.ento.53.103106.093301

[CR91] Wojcik DP (1994) Impact of the red imported fire ant on native ant species in Florida. In: Williams DF (ed) Exotic ants: biology, impact, and control of introduced species. CRC Press, pp 269–281

[CR92] Zulaikha AS, Zuha RM (2016) Nocturnal oviposition of the forensic scuttle fly, *Megaselia scalaris* (Loew) (Diptera: Phoridae) indoors. Egypt J Forensic Sci 6(4):489–491. 10.1016/j.ejfs.2016.02.002

