# Supplementary material for: Infestation by Phorid Flies Disrupts Behavior and Immune Function in Honey Bees Monitored by Radio-frequency Identification
Source: Neotrop Entomol. 2026 Feb 19;55(1):12. doi: 10.1007/s13744-025-01352-9 (PMC12920760; doi:10.1007/s13744-025-01352-9)
Supplement: Supplementary file 1 — Supplementary file1 (DOCX 2.27 MB) [file 13744_2025_1352_MOESM1_ESM.docx]

**Infestation by Phorid Flies Disrupts Behavior and Immune Function in Honey Bees Monitored by Radio-frequency Identification**

Gloria Ruiz-Guzmán^1,2^, Oliverio Delgado-Carrillo^1^, Francisco J. Balvino-Olvera^1,3^, María de Jesús Aguilar-Aguilar^1^, Violeta Patiño-Conde^1^, Paulo de Souza^4^, Ulises Olivares-Pinto^5^, Mauricio Quesada^1,6,*^

^1^Laboratorio Nacional de Análisis y Síntesis Ecológica (LANASE), Escuela Nacional de Estudios Superiores (ENES) Unidad Morelia, Universidad Nacional Autónoma de México (UNAM), Morelia, Michoacán, México.

^2^Posgrado en Ciencias Biológicas, Unidad de Posgrado, Ciudad Universitaria, Coyoacán, México.

^3^Unidad Académica de Ecología y Biodiversidad Acuática, Instituto de Ciencias de Mar y Limnología, Universidad Nacional Autónoma de México (UNAM), Ciudad de México, México.

^4^School of Engineering, Edith Cowan University, Perth, WA 6027, Australia.

^5^Escuela Nacional de Estudios Superiores (ENES) Unidad Juriquilla, Universidad Nacional Autónoma de México (UNAM), Campus Juriquilla. Querétaro, México.

^6^Instituto de Investigaciones en Ecosistemas y Sustentabilidad (IIES), Universidad Nacional Autónoma de México (UNAM), Campus Morelia, Morelia, Michoacán, México.

***Corresponding author:** [mquesada@cieco.unam.mx](mailto:mquesada@cieco.unam.mx)

**Table S1** GenBank accession numbers of assembled sequences included in this study

| **Species** | **Internal ID** | **Gene** | **Accession number** |
| --- | --- | --- | --- |
| *Megaselia sp.* | GRMS2016P312 | 12s | ON493548 |
| *Megaselia sp.* | GRMS2016P112 | 12s | ON493549 |
| *Megaselia sp.* | GRMS2016M712 | 12s | ON493550 |
| *Megaselia sp.* | GRMS2016M212 | 12s | ON493551 |
| *Megaselia sp.* | GRMS2016H801 | COI | ON548185 |
| *Megaselia sp.* | GRMS2016H501 | COI | ON548186 |
| *Megaselia sp.* | GRMS2016H401 | COI | ON548187 |
| *Megaselia sp.* | GRMS2016H301 | COI | ON548188 |
| *Megaselia sp.* | GRMS2016H201 | COI | ON548189 |
| *Megaselia sp.* | GRMS2016H701 | COI | ON548190 |
| *Megaselia sp.* | GRMS2016H601 | COI | ON548191 |
| *Megaselia sp.* | GRMS2016H001 | COI | ON548192 |
| *Megaselia sp.* | GRMS2016P416 | 16s | ON493608 |
| *Megaselia sp.* | GRMS2016P316 | 16s | ON493609 |
| *Megaselia sp.* | GRMS2016P216 | 16s | ON493610 |
| *Megaselia sp.* | GRMS2016PP16 | 16s | ON493611 |
| *Megaselia sp.* | GRMS2016B616 | 16s | ON493612 |
| *Apis mellifera scutellata* | GRAM20165L01 | COI | ON495694 |
| *Apis mellifera scutellata* | GRAM20165E01 | COI | ON495695 |
| *Apis mellifera scutellata* | GRAM20164L01 | COI | ON495696 |
| *Apis mellifera scutellata* | GRAM20164E01 | COI | ON495697 |
| *Apis mellifera scutellata* | GRAM20163L01 | COI | ON495698 |
| *Apis mellifera scutellata* | GRAM20163E01 | COI | ON495699 |
| *Apis mellifera scutellata* | GRAM20162N01 | COI | ON495700 |
| *Apis mellifera scutellata* | GRAM20162L01 | COI | ON495701 |
| *Apis mellifera scutellata* | GRAM20162E01 | COI | ON495702 |
| *Apis mellifera scutellata* | GRAM20161L01 | COI | ON495703 |
